# Structure and Synthesis of Antifungal Disulfide β-Strand Proteins from Filamentous Fungi

**DOI:** 10.3390/microorganisms7010005

**Published:** 2018-12-27

**Authors:** Györgyi Váradi, Gábor K. Tóth, Gyula Batta

**Affiliations:** 1 Department of Medical Chemistry, Faculty of Medicine, University of Szeged, H-6720 Szeged, Hungary; toth.gabor@med.u-szeged.hu; 2 MTA-SZTE Biomimetic Systems Research Group, University of Szeged, H-6720 Szeged, Hungary; 3 Department of Organic Chemistry, University of Debrecen, H-4032 Debrecen, Hungary; batta@unideb.hu

**Keywords:** structure, antifungal protein, chemical synthesis, solid-phase peptide synthesis, native chemical ligation, disulfide bond, NFAP2, PAF

## Abstract

The discovery and understanding of the mode of action of new antimicrobial agents is extremely urgent, since fungal infections cause 1.5 million deaths annually. Antifungal peptides and proteins represent a significant group of compounds that are able to kill pathogenic fungi. Based on phylogenetic analyses the ascomycetous, cysteine-rich antifungal proteins can be divided into three different groups: *Penicillium chrysogenum* antifungal protein (PAF), *Neosartorya fischeri* antifungal protein 2 (NFAP2) and “bubble-proteins” (BP) produced, for example, by *P. brevicompactum*. They all dominantly have β-strand secondary structures that are stabilized by several disulfide bonds. The PAF group (AFP antifungal protein from *Aspergillus giganteus*, PAF and PAFB from *P. chrysogenum,* *Neosartorya fischeri* antifungal protein (NFAP)) is the best characterized with their common β-barrel tertiary structure. These proteins and variants can efficiently be obtained either from fungi production or by recombinant expression. However, chemical synthesis may be a complementary aid for preparing unusual modifications, e.g., the incorporation of non-coded amino acids, fluorophores, or even unnatural disulfide bonds. Synthetic variants up to ca. 6–7 kDa can also be put to good use for corroborating structure determination. A short overview of the structural peculiarities of antifungal β-strand disulfide bridged proteins will be given. Here, we describe the structural propensities of some known antifungal proteins from filamentous fungi which can also be prepared with modern synthetic chemistry methods.

## 1. Introduction

Fungal infections represent a globally increasing health problem. There is a competition between pathogenic microbes and the efficacy of host-defense mechanisms [1]. In most of the cases, the defense systems augmented with antimicrobial peptides (AMPs) are more effective [2]. However, immunocompromised patients can be fatally threatened even from low-virulence fungi. Resistance to current small-molecule therapies leads to the need of more effective, alternative antifungal agents. Besides the conventional application of triazoles or echinocandins [3], there is an increasing interest in proteins as potential lead compounds for the design of novel antifungal drugs [4,5]. There are up to date reviews on antimicrobial peptides, [6] and plant defensins [7]. The antimicrobial peptide database (APD), http://aps.unmc.edu/AP/main.php contains more than 3000 sequences [8], and among them 1084 antifungal peptides can be found. They may be classified as α, β, αβ, and non-αβ, depending on their constituent secondary structures of α-helices and/or β-strands. Among them 43 are β-strand peptides with an average sequence length of 36 amino acids, spanning 10–106 residues. There is no sharp distinction between peptide or protein categories of molecules with peptide bonds. Peptides are relatively short sequences and may contain unnatural amino acid residues, while proteins typically have more than 50 residues and exhibit more structural order. Preparation of peptides by biotechnology or production of proteins by chemical synthesis—far from size limits—are often not affordable, so the two methods complement each other.

A decade ago, a classification of small, disulfide-rich protein domains from the Protein Data Bank (PDB) resulted in ca. 3000 structures [9] (note that the nomenclature is sometimes misleading, e.g., human α-defensin-4, PDB code: 1ZMM, contains exclusively β-strands.) In that review the identified domains could be assigned to 41 different folds and 98 families of disulfide-rich domains were defined. The 43 antifungal, β-strand peptides are cationic and cysteine rich, with an average net charge of ca. 5 at neutral pH, which is provided by the high arginine and/or lysine content. Their fold must be stabilized by disulfide bonds, otherwise the electrostatic repulsion would result in disordered structure. Thirty-four of them were studied by Nuclear Magnetic Resonance (NMR), and only nine by X-ray crystallography. Fifteen of these proteins are secreted by filamentous ascomycetes.

Compared to these bewildering numbers, the structures here are limited (Table 1) and this review focuses on antifungal β-strand proteins. Among them there are the proteins belonging to the *Penicillium chrysogenum* antifungal protein (PAF) group, of which structures have been determined recently by NMR, while the bubble-protein (BP) structure was obtained by X-ray crystallography [10,11]. Knowledge of structure, dynamics and folding of these peptides and proteins is the first step towards the understanding of their mode of action.

In fact, the detailed mechanism of action in the antifungal proteins is not known at a molecular level. There is a consensus that, thanks to their amphipathic nature, they traverse the cell membrane without destroying it. PAF induces apoptotic cell death, probably after heterotrimeric G-protein coupled signaling, but toxicity may also be connected with disturbed cation homeostasis [14]. According to a recent hypothesis, the evolutionary conserved γ-core motif of approx. ten residues, e.g., in PAF [12], and in anti-fungal protein (AFP), can be a common sequence responsible for their antimicrobial activity.

In many cases, native proteins and their variants—even when equipped with ^15^N, ^13^C isotope labelling—can be prepared by recombinant expression. In the last two decades discovery of genetically encoded fluorescent tags and improvements in non-standard amino acid incorporation into proteins [15,16,17,18] have opened the way for the preparation of labelled or non-proteinogenic amino acid containing proteins by biotechnological methods. Nevertheless, recombinant protein expression has limitations, e.g., low yields in some host systems [19,20,21]. Alternatively, chemical synthesis can be used as a complement to biotechnology (Table 2). A byproduct of the chemical synthesis of native proteins is that it may help to verify structural details, e.g., to find the right disulfide pattern, that is sometimes difficult to prove by other means, e.g., by NMR.

The scope of this review is to cover recent progress in structure determination and synthetic chemistry of antifungal β-strand, disulfide proteins.

## 2. Structure Determination of Antifungal Disulfide β-Strand Proteins from Filamentous Fungi

Structure determination of small proteins by X-ray crystallography [8] is generally not straightforward because of crystallization difficulties. Until now, besides some smaller peptides, the X-ray structure of the bubble protein in the antifungal protein family was published [11], PDB code: 1uoy. It has a globular, all-β secondary structure (except a short helix) with a new fold, and surface electrostatic charge similar to the *Williopsis mrakii* killer toxin. The advantage of X-ray structure is the unambiguous disulfide pattern and conformation determination.

**Figure 1:**
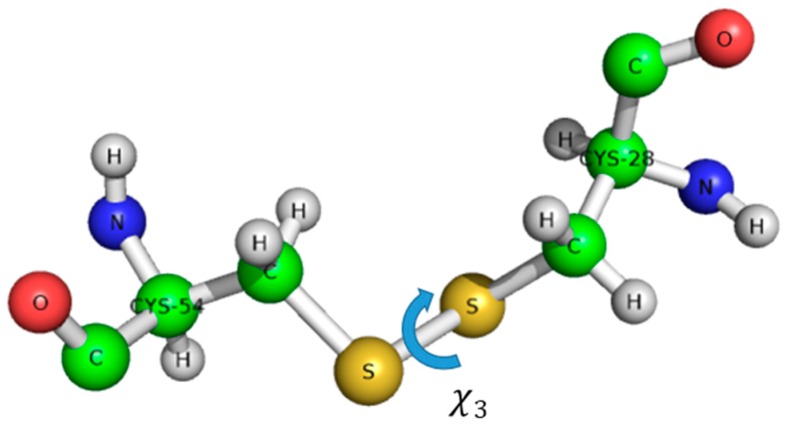
Definition of χ_3_ dihedral angle on the example of *Penicillium chrysogenum* antifungal protein (PAF) Cys28-Cys54 disulfide bond.

The structure of AFP was solved by ^1^H-NMR, without isotope labelling [29]. AFP is the structural ancestor of the PAF family, possessing a Greek key supersecondary structure of antiparallel β-strands connected by four loops, resulting in a β-barrel tertiary structure. The most probable disulfide bond pattern in AFP is “abcdabcd”, connecting cysteines 7–33, 14–40, 26–49, and 28–51 [30]. There are ambiguities, and a minor AFP variant was also detected with a possibly different, though unknown disulfide pattern and structure. Unfortunately, these proteins often resist against proteolytic digestion for unambiguous MS disulfide pattern determination, and are stable even under harsh stress conditions (pH, temperature, chemical denaturants, and pressure). Another complicating factor could be the possible disulfide shuffling (switching of disulfide pattern) or conformational exchange within the individual disulfide bonds, that might even result in chirality changes [31,32]. The disulfide bonds have helical chirality; the dihedral angle Χ_3_ can be +90° or –90°. The full conformation is described by five dihedral angles Χ_1_, Χ_2_, Χ_3_, Χ_1’_, Χ_2’_, that determine the energy of the disulfide bond (Figure 1) [33].

**Figure 2:**
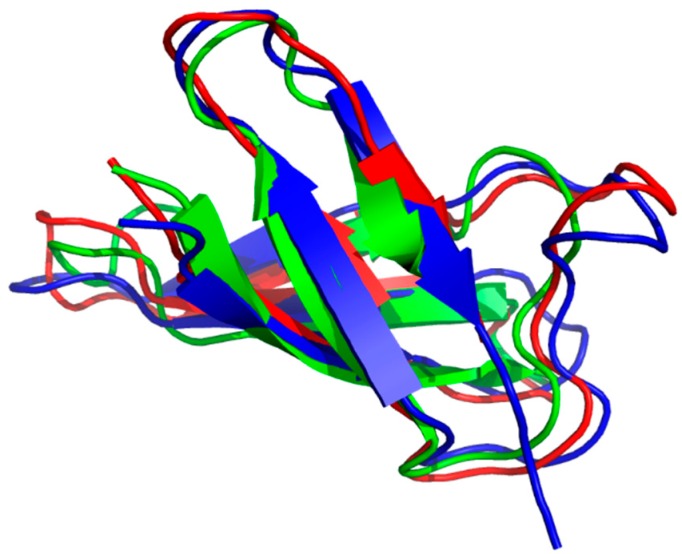
Triple alignment of anti-fungal protein (AFP) (1afp), PAF (2mhv) and *Neosartorya fischeri* antifungal protein (NFAP) (5oqs) along their CA atoms. (Pymol software tool). Antiparallel β-strands represented by arrows form two overlapping β-sheets. Consecutive strands are connected by short turns or longer loop regions. Green color stands for AFP, red for PAF and blue for NFAP.

A recent work [34] suggests that the disulfide conformation can be determined directly from NMR chemical shifts. A good example of the progress of a disulfide protein structure is PAF. Using ^15^N labelled PAF, the NMR solution structure has been determined (PDB code: 2kcn). The β-barrel fold was correctly established [35], and exhibited high structural homology with AFP. In addition, ^15^N-NMR relaxation measurements have proven a rather rigid structure based on S^2^ order parameters. Structural ensembles were calculated with the Minimal Under-restraining Minimal Over-restraining (MUMO) method using S^2^ parameters as dynamical constraints. The measured NH deuteration rates and Chemical Shift Anisotropy/Dipole-Dipole (CSA/DD) (^15^N chemical shift anisotropy - ^15^NH dipolar relaxation) interference terms were in accordance with the proposed secondary structure. A more detailed NMR study [36] using ^13^C-^15^N labelled PAF allowed further insight into the structural and dynamical behavior. The new structure (PDB code: 2mhv) corroborated the earlier fold with better precision. Co-crystallization of PAF with sulfonato-calixarenes resulted in an X-ray structure of PAF [37] in perfect agreement with NMR. Interestingly, PAF exhibits reversible cold and heat unfolding in a broad temperature range. Even sporadic conformers (0.15% population and very different from the native structure) could be observed by NMR Chemical Exchange Saturation Transfer (CEST) technique. The inactive PAF-D19S was also shown to have similar sporadic conformers. (In PAF-D19S variant, the aspartic-acid residue in position 19 has been replaced by serine.) In that case, the loss of activity was suspected due to reorganized electrostatic surface of a very similar structure to PAF or lack of unfolding intermediate states [38]. In a recent paper, it was shown that the structure of sfPAFB (PDB code: 2NC2) exhibits high structural homology with PAF, in spite of low (35.2%) sequence similarity [39]. The sfPAFB sequence means “short form” of PAFB, without the first two residues at the N-terminus of PAFB. The structure of NFAP was published recently (PDB code: 5oqs) (Figure 2). It seems that the members of the PAF cluster have high structural similarity, while NFAP2 [40] and bubble proteins [11] are different both in structure and dynamics.

It is expected that determining more details on the structure and dynamics of antifungal disulfide proteins will aid future design of similar agents, and hopefully lead to better understanding of their very selective mode of action.

## 3. Chemical Synthesis of Peptides and Proteins

### 3.1. Solid-Phase Peptide Synthesis

As discussed in the Introduction session, peptides and proteins are structurally very similar: both are polymers of amino acids. In point of chemical synthesis, the main distinguishing feature of them is the size: Proteins are bigger than peptides. (An arbitrary distinction between them is about 50 amino acids.) Both peptides and smaller proteins can be prepared by solid-phase peptide synthesis (SPPS). This revolutionary technique of Merrifield is the stepwise construction of peptides and proteins on an insoluble, pre-functionalized polymeric support called resin [41]. Apart from solution-phase synthesis, unreacted reagents (and also by-products) can be removed by washing. It allows the usage of excess of reagents, and thus drives the reaction to completion. Moreover, repeated cycles of the solid-phase method can be automated. The most modern microwave-assisted peptide synthesizers operate with very short cycle times (just a few minutes), and the length of the peptide or protein chain is increased by one amino acid in each cycle.

Reactive functional groups of the applied amino acids should be protected during the synthesis. Based on the temporary protecting group of α-amino groups, there are different strategies for SPPS. The most widely used strategy is based on Fmoc (9-fluorenylmethoxycarbonyl), a base-labile N^α^-protecting group. It utilizes *t*-butyl-based side-chain protection for amino acids. After completion of the synthesis, mild acidolysis detaches the peptide or protein from the resin and cleaves off the side-chain protecting groups. Synthesized peptides and proteins are analyzed by mass spectrometry to confirm the correct molecular mass. Their purity is checked by analytical Reversed-Phase High-Performance Liquid Chromatography (RP-HPLC). Crude peptides or proteins can be purified by RP-HPLC, if necessary.

### 3.2. Native Chemical Ligation

**Figure 3:**
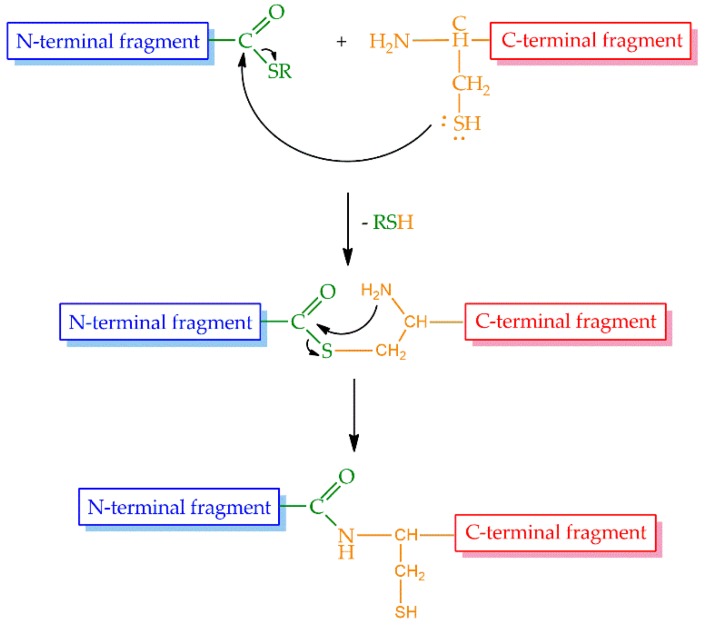
The process of native chemical ligation.

Even though the above-mentioned cycles of the SPPS could be repeated theoretically without limitations, in practice, the size of the peptides or proteins that could be prepared by stepwise synthesis was limited to approximately 50 amino acid residues, and this size was sequence dependent. Therefore, a large demand existed for the possibility to increase the size of peptides and proteins synthesized by chemical methods. The most promising approach was the introduction of native chemical ligation (NCL) by Kent and coworkers in 1994 [42]. The strategy is based on a reversible thioester exchange between a C-terminal peptide thioester and an N-terminal cysteine of another peptide yielding thioester intermediate. It undergoes a rapid and irreversible intramolecular rearrangement producing a native peptide bond between the two fragments (Figure 3).

The extension of NCL allows ligation in some amino acids other than cysteine. In the lack of cysteine, auxiliary mediated ligation (AML) [43] or desulfurization after NCL can be applied [44]. AML is a method in which a free thiol of an auxiliary group at the N-terminus reacts with the thioester. Several auxiliary groups were developed in the last two centuries allowing ligation in various amino acids and cleavage of the auxiliary under different conditions. Desulfurization after NCL can be a method of choice if a peptide having alanine, phenylalanine, lysine or valine at its N-terminus should be ligated with another fragment. Beside these extensions, the so-called sequential ligation permits ligation of three or more peptide fragments, and thus, the preparation of even bigger proteins.

### 3.3. Formation of Disulfide Bonds

Disulfide bonds play a key role in folding and structure stabilization of several peptides and proteins [35,45,46,47]. (The structures of antifungal proteins from filamentous fungi are stabilized by three or four disulfide bridges). Disulfide bridges can be formed in the last step of synthesis. The major problem arising during disulfide bond formation in chemical systems is the possibility of the formation of numerous different regioisomers. In the case of six cysteines, 15, and in case of eight cysteines, 105 different isomers are possible. In nature, well-defined redox mechanisms control the formation of the correct, biologically active disulfide motifs [48]. In addition to that, there is an enzyme in the endoplasmic reticulum, the protein disulfide isomerase [49,50,51], which catalyzes the trans isomerization of the wrong folds into the correct, biologically active isomer [52]. Unfortunately, everything is much more complicated during chemical synthesis. The primary sequence more or less determines the steric positions of the cysteines and in consequence the formation of the thermodynamically preferred disulfide pattern, but several wrong patterns can be formed. Therefore, the formation of the correct disulfide pattern in case of synthetic biopolymers is a challenging task even today.

Chemical strategies for the formation of disulfide bonds are based on the thiol protecting groups of cysteines. It is possible to protect side chains of cysteines either identically or orthogonally. In the former case, disulfide bridges can be formed by the oxidation of free thiols of cysteines in a diluted, and usually slightly basic, solution. The simplest method is the application of oxygen in air by intensively stirring the solution. Sometimes a reducing agent, such as cysteine or reduced glutathione, helps to refold the wrong disulfide isomers into the desired, biologically active form [53,54]. If it is necessary, chaotropic agents (e.g., urea, guanidinium chloride, ethanol, n-butanol, sodium dodecyl sulfate) may be used to solubilize the peptides or proteins, and prevent their aggregation. In the latter case, thiols of cysteines are protected by orthogonal protecting groups, and disulfide bridges are formed in a regioselective manner. Although this strategy seems to provide an unambiguous solution to the formation of a desired disulfide bond pattern, in practice some reagents used for the cleavage of thiol protecting groups open up the previously formed –S-S- bonds, and thus, lead to wrong isomers.

## 4. Application of Chemical Methods for the Preparation of Antifungal Proteins from Filamentous Ascomycetes

The family of antifungal disulfide β-proteins from filamentous fungi is small, only 15 members were isolated and (partially) characterized up to now [12]. The number of proteins, and also the number of research groups working on this field, are very limited. In addition, biological methods such as isolation from natural sources or heterologous expression, can be efficiently used for the production of proteins with the native structure and some variants of them. Only two of them were synthesized by chemical methods up to now: PAF [46] and NFAP2 [40].

The structures of PAF and NFAP2 are similar regarding the number of amino acids and disulfide bonds. PAF is a 55-mer and NFAP2 is a 52-mer peptide having six cysteine residues in their sequences. Both structures are stabilized by three disulfide bridges. Containing more than 50 amino acids, stepwise synthesis was not a matter of choice for their preparation. They were synthesized from two fragments by native chemical ligation of the parts. The last step of the synthesis, formation of natural disulfide bond pattern, could be carried out under different conditions in both cases.

### 4.1. Synthesis of PAF

**Figure 4:**
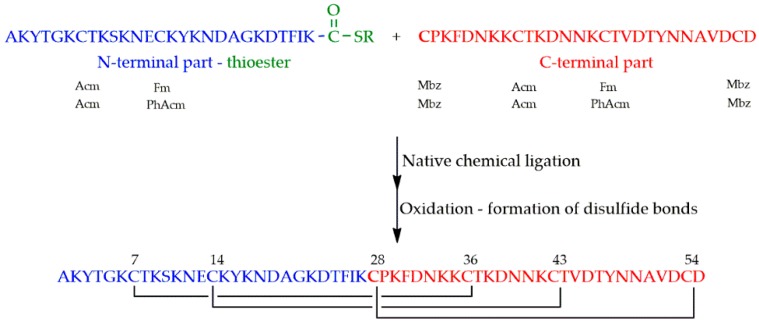
Synthesis of PAF by native chemical ligation and subsequent oxidation. (Thioester is symbolized by COSR). Orthogonal protecting groups of cysteines (shown below the sequences) were as follows: Acetamidomethyl (Acm), fluorenylmethoxycarbonyl (Fm), phenylacetamidomethyl (PhAcm) and methylbenzyl (Mbz).

PAF was synthesized from the thioester of a 27 amino acid containing N-terminal and a 28 amino acid containing C-terminal parts having cysteine at its N-terminal end (Figure 4). A newly discovered polymer (Cys-SH resin) was used for the preparation of the thioester [46].

As a first attempt, thiol functions of cysteines were protected orthogonally to ensure the formation of disulfide bonds in a regioselective manner. Two different sets of protecting groups have been tried out: *p*-methylbenzyl (Mbz), acetamidomethyl (Acm) and fluorenylmethyl (Fm), and *p*-methylbenzyl (Mbz), acetamidomethyl (Acm) and *S*-phenylacetamidomethyl (Phacm). In the former case, basic treatment, used for the cleavage of Fm, rearranged the previously formed disulfide bonds. In the latter case, neither the immobilized, nor the dissolved form of penicillin G amidase enzyme (EC 3.5.1.11) managed to detach Phacm.

As second attempt, identical protecting groups (Mbz) were introduced for the six cysteines. The crucial step—formation of right pairing of cysteines—was performed with the use of an oxidizing and a reducing agent together. Two systems were found to be appropriate: (1) oxygen of air together with cysteine and (2) a glutathione redox system. Folding was performed in a 0.1 M ammonium acetate buffer at pH 7.5 applying 0.2 mgmL^−1^ protein concentration.

### 4.2. Synthesis of NFAP2

**Figure 5:**
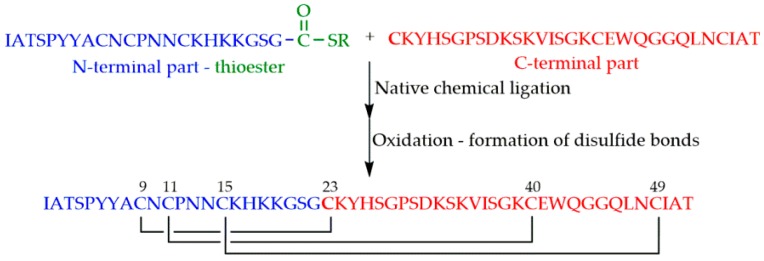
Synthesis of *Neosartorya fischeri* antifungal protein 2 (NFAP2) by native chemical ligation and subsequent oxidation.

NFAP2 was synthesized by chemical ligation of the N-terminal 22-mer and the C-terminal 30-mer fragments [40] (Figure 5). Thioester of the N-terminal part was prepared on the previously mentioned Cys-SH polymer.

Identical protection (Mbz) was used for thiols of cysteines. A glutathione redox buffer containing both the oxidized and the reduced forms of glutathione was found to be appropriate for folding and formation of natural disulfide bond pattern.

## 5. Summary

Highly efficient production of antifungal proteins from filamentous fungi or their variants have become available recently from wild type or mutant strains, and sometimes by heterologous expression. Their tertiary structures are solved by NMR or X-ray crystallography, possibly equipped with in silico methods. The alternative, chemical synthesis of the proteins discussed here helps to disclose possible structural ambiguities, e.g., disulfide patterns. Moreover, synthesis of their short peptide segments with putative antifungal activity, or construction of unnatural analogues are invaluable for structure-function studies. In addition to structural overview, this mini-review discusses recent synthetic methods for preparing antifungal proteins: Solid-phase synthesis and native chemical ligation, and covers efficient strategies for building the required disulfide bridges. Some practical aspects of the synthesis are demonstrated in case of two antifungal proteins from filamentous ascomycetes.

## Figures and Tables

**Table 1:** Classification of antifungal β-strand, disulfide proteins. Mw and pI were calculated with ProtParam (https://web.expasy.org/cgi-bin/protparam/protparam). In residue statistics n*K and n*G stand for number of lysine and glycine residues. GRAVY hydropathy scores are calculated via (http://www.gravy-calculator.de). Lower GRAVY values mean less hydrophobic structures. NFBP is *Neosartorya fischeri* bubble protein [12].

	PAF Cluster	NFAP2 Cluster	BP Cluster
**Representatives**	AFP, PAF, PAFB, NFAP	NFAP2	BP, NFBP [12,13], Pc-Arctin [13]
**Number of residues**	51, 55, 58, 57	52	64, 64, 64
**Approximate Mw (kDa)**	5.8, 6.3, 6.5, 6.6	5.6	6.6, 6.8, 6.6
**Number of disulfide bonds**	4, 3, 3, 3	3	4, 4, 4
**Most abundant residues**	12*K, 13*K, 8*K, 11*K	7*K and 6*G	13*G, 11*G, 14*G
**Isoelectric point (pI)**	9.3, 8.9, 8.8, 8.9	9.0	7.7, 6.9, 7.7
**GRAVY score from sequence**	–0.91, –1.4, –1.03, –1.21	–0.73	–0.87, –0.96, –0.77
**Experimental structure**	β-barrel all, (NMR)	unknown	αβ (X-ray), unknown
**Biological effects**	anti-mold, anti-yeast, antiviral	anti-yeast	anti-mold, anti-yeast

**Table 2:** Possibilities of protein production by biotechnology and chemical synthesis.

	Recombinant Technology	Chemical Synthesis
**Size limit**	Theoretically unlimited.	Stepwise synthesis: ~50 amino acids (sequence dependent). Using ligation: Theoretically unlimited, but technically and financially demanding.
**Posttranslational modification**	Possible [22].	Possible with limitations [23].
**Unusual modifications**	Strongly limited. Recent discoveries: Genetically encoded fluorescent tags [24,25,26], incorporation of non-standard amino acids [15,16,17,18], synthesis of selenocysteine-containing [27] or fluorinated [28] compounds for NMR applications.	Possible.
**Application for antifungal disulfide β-strand proteins**	Native disulfide pattern is expected.	Selective (arbitrary) disulfide bond formation is possible.
